# Automated Chemotactic Sorting and Single-cell Cultivation of Microbes using Droplet Microfluidics

**DOI:** 10.1038/srep24192

**Published:** 2016-04-14

**Authors:** Libing Dong, Dong-Wei Chen, Shuang-Jiang Liu, Wenbin Du

**Affiliations:** 1 Department of Chemistry, Renmin University of China, Beijing 100872, China; 2 State Key Laboratory of Microbial Resources, Institute of Microbiology, Chinese Academy of Sciences, Beijing 100101, China

## Abstract

We report a microfluidic device for automated sorting and cultivation of chemotactic microbes from pure cultures or mixtures. The device consists of two parts: in the first part, a concentration gradient of the chemoeffector was built across the channel for inducing chemotaxis of motile cells; in the second part, chemotactic cells from the sample were separated, and mixed with culture media to form nanoliter droplets for encapsulation, cultivation, enumeration, and recovery of single cells. Chemotactic responses were assessed by imaging and statistical analysis of droplets based on Poisson distribution. An automated procedure was developed for rapid enumeration of droplets with cell growth, following with scale-up cultivation on agar plates. The performance of the device was evaluated by the chemotaxis assays of *Escherichia coli* (*E. coli*) RP437 and *E. coli* RP1616. Moreover, enrichment and isolation of non-labelled *Comamonas testosteroni* CNB-1 from its 1:10 mixture with *E. coli* RP437 was demonstrated. The enrichment factor reached 36.7 for CNB-1, based on its distinctive chemotaxis toward 4-hydroxybenzoic acid. We believe that this device can be widely used in chemotaxis studies without necessarily relying on fluorescent labelling, and isolation of functional microbial species from various environments.

This paper describes a simple microfluidic assay which can evaluate chemotactic responses of non-labelled microbial cells, and can directly isolate and cultivate microbial cells with high chemotactic mobility from pure cultures or mixtures. Chemotaxis is a remarkable characteristic of microbial cells^1^,^2^, which allows them to migrate toward favorable environments and escape from hazardous substances. It plays essential roles in nutrient acquisition^3^, biofilm dispersal^4^, bacterial-host interaction^5^, and pathological mechanisms of infectious diseases^6^. The chemotactic motility may also provide a species with competitive advantages compared with non-chemotactic species, which result in higher diversity of motile species in the environments^7^.

Significant efforts have been devoted to the development of assays for investigating microbial chemotaxis since 1960 s^8^. Among them, swarm plate assays^9^ and capillary assays^10^ are widely recognized for their simplicity and convenience, and feasibility of label-free evaluation of chemotaxis at population level. Recently, various microfluidic devices for chemotaxis assays have been introduced^11^,^12^, which could be classified as either flow-based or flow-free strategies^11^. In flow-free methods, time-varying gradients are established based on diffusion in microstructures^13^. Materials such as hydrogels and porous membranes can be incorporated to create steady gradients^14^,^15^. Flow-free methods provide well-controlled gradients for chemotaxis in the absence of flow, and trajectories of individual cells in the gradients can be traced by imaging. Instead, flow-based methods use parallel-flow to generate chemoeffector gradients across the channel^16^,^17^. An important advantage of flow-based methods is that it allows continuous collections of cells with branched outlets, which facilitate chemotaxis-based cell sorting from mixtures.

Most microfluidic devices rely on invasive labelling to visualize microbial cells under microscope. Cells are usually transformed with fluorescent protein plasmids, or strained with fluorescent dye prior to the assays. These processes inevitably perturb the physiological condition of cells including its motility and chemotactic response^18^. For many environmental and anaerobic microbial species, fluorescent labeling are very difficult or impossible. Although a recent study has reported the use of phase contrast microscopy^14^ in visualization of chemotaxis in flow-free gradients, it is still challenging to evaluate chemotaxis without labelling of cells.

Microfluidic technique for generating mono-disperse droplets spaced by immiscible carrier oil has attracted substantial interests in the past few years. The superiorities of high throughput screening and sorting in picoliter to nanoliter volumes^19^ have made it successfully applied in a wide range of areas including digital quantification of nucleic acids^20^,^21^, crystallization screening^22^, and single-cell analysis^23^,^24^,^25^,^26^,^27^. One of essential utilities of droplets is to serve as micro-compartments for isolating single cells for growth, reaction or detection^28^,^29^. The random distribution of cells in a large number of droplets allows separation of different species, as well as precise counting of cell numbers after incubation^30^,^31^. In addition, single-cell isolation in droplets has been validated that it can improve recovery of slow-growing species^32^,^33^.

In this work, we introduce a microfluidic system which interfaces parallel-flow based chemotactic sorting with droplet microfluidics. This system takes advantage of both parallel-flow and droplet microfluidics. Steady-state gradient for chemotactic sorting and high throughput droplet encapsulation for single cell cultivation are incorporated in a seamless and automated manner. The use of droplets allows straightforward enumeration of non-labelled cells for quantitative evaluation of chemotaxis, and also enable enrichment and rapid recovery of species for further studies.

## Results and Discussion

### Design of the microfluidic system

**Figure 1:**
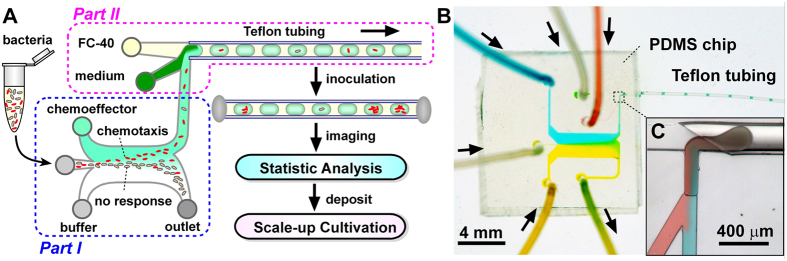
(**A**) Schematic of the device for chemotactic sorting and droplet cultivation; (**B**) Photograph of a device filled with food dye solutions. (**C**) Zoom-in view of food dye droplet generation on the device.

The microfluidic device was designed to interface a chemotactic cell sorter (*Part I*) with droplet-based single-cell encapsulation system (*Part II*) for continuous chemotactic sorting and cultivation (Fig. 1). *Part I* contains three inlets, a main channel for chemotactic migration of cells, and two asymmetric outlet channels. The depth of channels in *Part I* is 100 μm. The main channel is 5 mm long. The cell inlet is 100 μm in width, each buffer inlet is 1.0 mm in width, and the total width of the channel is 2.1 mm. The gradient for chemotaxis in the main channel was developed among three streams by parallel-flow diffusion. Cells swam from the middle stream to the upper region following the direction of the gradient. *Part II* includes two inlets, a T-junction droplet generator, and the Teflon tubing (200 μm I.D., 250 μm O.D., Zeus, Branchburg, NJ) for collecting droplets. The depth of channels in *Part II* is 200 μm. Cell suspension was merged with culture medium, and segmented into nanoliter droplets by the shear flow of carrier oil (FC-40). In our experiments, Teflon tubing with 30-cm length can store about 100~200 droplets. After the tubing was filled with droplets, it was detached from the device, sealed at both ends with wax and cultivated. The gas-permeable Teflon tubing and carrier oil supported growth of aerobic species in the droplets. Chemotactic response was assessed by counting of droplets containing cell growth, following with statistical analysis based on Poisson distribution.

### Building chemical gradient using parallel-flow

**Figure 2:**
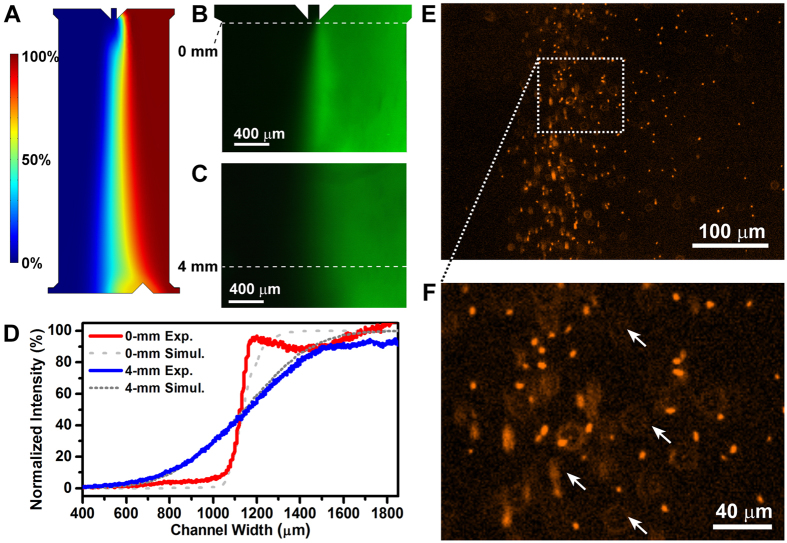
Chemotaxis assays based on parallel-flow. (**A**) Numerical simulation of diffusion gradient at the interface of parallel-flow in the main channel, assuming a total flow rate of 0.53 μL/min. (**B,C**) Fluorescent imaging of the diffusion gradients for fluorescein in the main channel, at the inlet and 4 mm downstream from the inlet. (**D**) Experimental validation of cross-sectional diffusion profiles at 0 mm (red line) and 4 mm (blue line) below the junction were compared with theoretical simulations. (**E**) Representative fluorescent image showing response of *E. coli* RP437 to the gradient of 10 mM aspartic acid (Asp). (**F**) Zoom-in view shows that some cells were not clearly imaged (indicated by the white arrows). The experiments were performed at a total flow rate of 0.53 μL/min.

We simulated the laminar flow and diffusion in the main channel by COMSOL Multiphysics (COMSOL, Inc., Burlington, MA, USA), using fluorescein as a model “chemoeffector”. Initial flow rates for chemotaxis assay were defined as: 0.25 μL/min for two side inlets, 0.025 μL/min for the middle inlet, and 0.25 μL/min for the left outlet. The diffusion coefficient of fluorescein (MW 372) in water (4.9 × 10^−6^ cm^2^/s) was used. The gradient evolved from a narrow and steep step at the junction to a smooth and wide slope at the downstream (Fig. 2A). Experiments were performed following the same condition with 100 μM fluorescein in 1 × PBS introduced at the right inlet, and buffer introduced at the left and middle inlets. The corresponding fluorescent intensities at the junction of three inlets and 4 mm downstream were imaged and the intensity profiles were recorded (Fig. 2B,C). The experimental and theoretical profiles at these two positions were compared, indicating a good match between the experiment and simulation (Fig. 2D).

### Imaging of microbial chemotaxis in parallel-flow

The flow-based gradient allows us to load and trigger chemotactic responses of bacteria cells toward various chemoeffectors rapidly. To investigate whether the chemotactic responses of cells toward the flow-based gradient in the main channel can be directly assessed, we carried out an experiment in which RFP-tagged *Escherichia coli* (*E. coli*) RP437 was exposed to the gradient of aspartic acid (Asp) in the main channel. Fluorescent images were captured every 10 s duration 10 min. Fluorescence imaging demonstrated that *E. coli* RP437 cells were skewed toward the side of Asp. Analysis of sequences of images yielded the difference of cell distributions arising from chemotactic response (Fig. 2E,F). It should be noted that the fluid flow in the main channel fell in laminar regime, with a highest velocity at the center of the channel and approaching to zero near the wall region^34^. We tested total flow rates from 0.53, 1.05 to 2.10 μL/min, and the concentration of Asp including 0.2, 1.0, 5.0, 10, and 20 mM. The optimal condition for chemotaxis of RP437 was selected at the flow rate of 0.53 μL/min and 10 mM Asp (Fig. S1). At the optimal total flow rate of 0.53 μL/min, the average velocity was about 42 μm/s. As the fluorescence from the *E. coli* RP437 cells (0.3~1 μm in diameter, ~2 μm in length) was very limited, the optimal exposure time (200 ms) for fluorescent imaging was chosen, leading to a theoretical displacement of 8.4 μm during each exposure. Therefore, we could not clearly capture a large portion of cells (Fig. 2F), thus affected the accuracy of flow-based chemotaxis assays on this device. Nevertheless, the real time visualization of chemotaxis in the main channel helped us to quickly optimize the experimental conditions according to specific chemotaxis properties of the tested species.

### Evaluation of chemotaxis based on droplet cultivation

**Figure 3:**
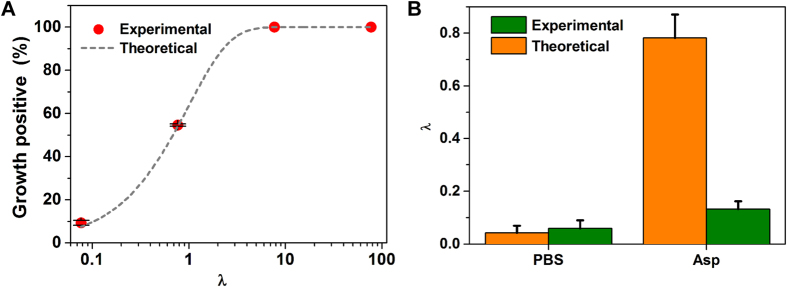
Evaluation of chemotaxis of microbial cells based on nanoliter droplet cultivation. (**A**) Percentages of cell-containing droplets in array for serial diluted cell suspensions. *E. coli* RP437 was used as the sample to direct mix with culture media to form droplets. The experimental data is in good agreement with theoretical predication (black dotted curve). (**B**) Difference of the average number of cells per droplet (*λ*) for *E. coli* RP437 and *E. coli* RP1616 with and without 10 mM Asp as the chemoeffector. A 1:1 mixture of *E. coli* RP437 and *E. coli* RP1616 was used as the sample.

Microfluidic-generated droplets had been proposed for precise measurements of the concentration of cells or molecules^30^,^35^. To evaluate the precision of cell density measurement based on Poisson distribution, we prepared a series of cell suspensions of *E. coli* RP437 with different cell density, and mixed them with culture medium to form droplets with cell densities from 0.6 × 10^3^ to 0.6 × 10^6^ CFU/mL. The droplets were stored in Teflon tubing and incubated to allow cell growth. Total number and number of droplets with cells were enumerated to perform the statistical analysis. The results show that cell distribution in droplets fits well with the theoretical predication (Fig. 3A).

Cells at the outlet of the main channel were split into two streams: those showed strong chemotactic responses were interfaced with the droplet generator for cultivation, and others were discarded through the waste outlet. For proof-of-concept, *E. coli* RP437 and *E. coli* RP1616 were tested for their chemotactic responses to Asp. *E. coli* RP1616 is a mutant of *E. coli* RP437 which loses chemotactic ability due to deletion of *cheZ* gene^13^. After the samples were exposed to the Asp gradient in the main channel, the upper stream containing chemotactic cells was mixed with culture media, and split to form uniform droplets. The optimal flow rates of cells, culture media, and the carrier oil were 0.25 μL/min, 0.3 μL/min and 3.0 μL/min, respectively, yielding monodisperse droplets with an average volume of 13 nL at a frequency of ~40 drop/min continuously. Droplet size and frequency depended on properties of the culture media and flow rates, and remained constant in each experiment. Other experimental settings were the same as above. The droplets were incubated at 37 °C for 24 h to allow growth of cells.

Microbial growth in droplets was observed by bright-field and fluorescence microscopy (Fig. S2A). For each experiment, ~100 droplets were analyzed to determine the mean number of cells per drop based on Poisson distribution. The average number of *E. coli* RP1616 cells per droplet remained low when exposed to Asp, compared with the control experiment with 1 × PBS (Fig. 3B, Fig. S2B,C). In contrast, the cell density per drop of *E. coli* RP437 increased from 0.043 to 0.77. This result indicates that droplet encapsulation is suitable for evaluation of microbial chemotaxis.

### Automated enumeration of droplets with cell growth

**Figure 4:**
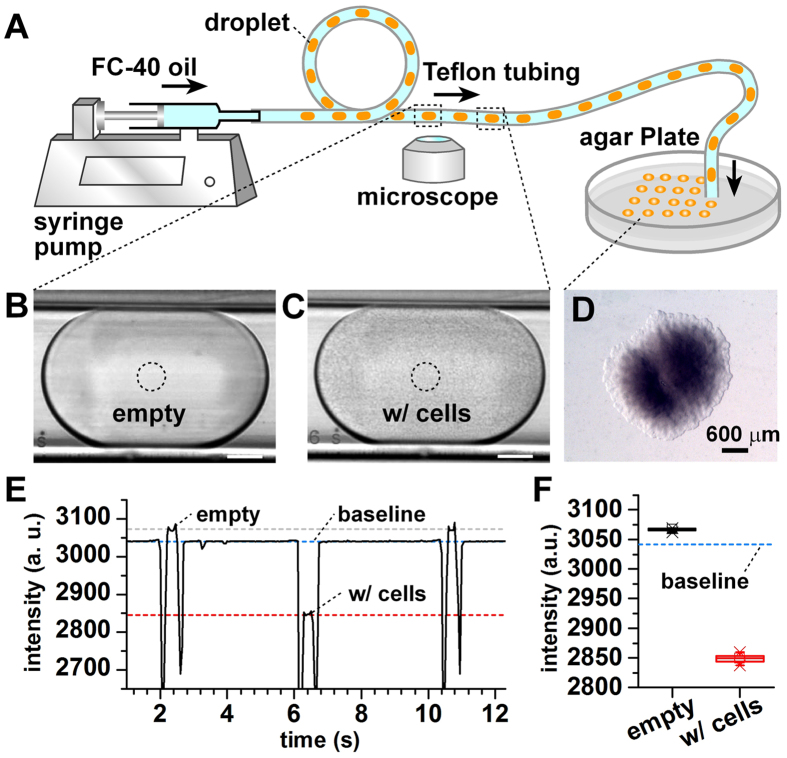
Rapid enumeration of droplets with cell growth. (**A**) Schematic of automated droplet array detection and scale-up cultivation of the droplet array. (**B,C**) Typical bright-field microscopic images of empty droplets (**B**) and cell-containing droplets (**C**). *E. coli* RP437 was used as the cell sample. Scale bar = 50 μm. (**D**) Colony growth of *E. coli* RP437 on agar plate inoculated with a single droplet. (**E**) Distinguish empty and cell-containing droplets using bright-field (BF) video recording. (**F**) Box plot of BF intensities for empty droplets and cell-containing droplets.

We introduced a simple workflow for automated classification of droplets, following with deposition of droplets on agar plates for scale-up cultivation. The Teflon tubing with the droplet array was connected with a syringe pump to infuse the droplets through the focal plane of the microscope at a flow rate of 0.5 μL/min (Fig. 4). Bright-field images of all droplets, either empty or cell-containing, were obtained by video recording at 16.8 fps (Fig. 4B,C). During the imaging process, the outlet of the tubing was operated manually to streak droplets on agar plates^33^. The speed of manual streaking was optimized to avoid depositing more than one droplet per location. The plates were incubated to allow colony growth (Fig. 4D), following recovery of isolates for further study and characterization. The average intensity in the center of the tubing where the droplets passed through was measured with the recorded images, and bimodal profiles were obtained for all droplets (Fig. 4E). The signal of each droplet can decompose into a plateau (corresponds to the homogeneous droplet signal), and two narrow peaks (corresponds to front and back boundaries of the droplet). The signal amplitude for each droplet was derived from the height of the plateau. The results reveal that the intensity for droplets containing cell growth was significantly lower than empty droplets, due to optical absorbance (Fig. 4E,F).

### Non-labelled chemotaxis of pure cultures

**Figure 5:**
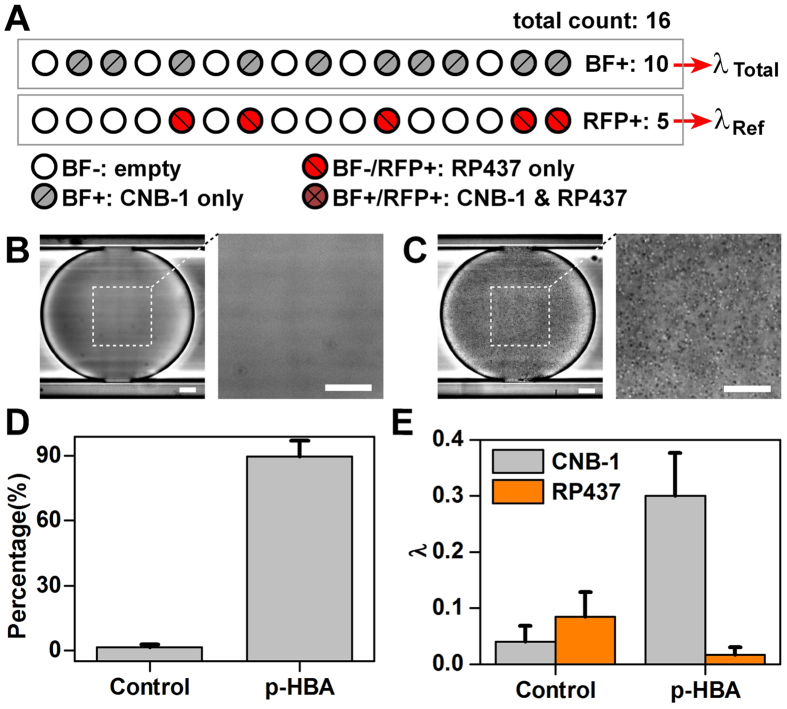
Separation of non-labelled chemotactic cells from mixtures. (**A**) Calibration of chemotaxis of non-labelled species with a fluorescent labelled reference strain. Droplets are imaged using both bright-field and fluorescence microscopy, and classified as empty, only reference strain, only non-labelled strain and both strain. The cell density of non-labelled strain was analyzed based on Poisson distribution. (**B,C**) Phase-contrast microscopic images of droplets in Teflon tubing after 24 h cultivation: (**B**) empty, (**C**) with growth of non-labelled CNB-1. Scale bar = 25 μm. (**D**) Evaluation of the chemotaxis of non-labelled CNB-1 using droplet-based enumeration, comparing before and after exposure to *p*-HBA with the same experimental setting. (**E**) Chemotactic sorting of non-labelled CNB-1 from its 1:10 mixture with *E. coli* RP437, comparing before and after exposure to *p*-HBA.

To investigate if our system can be used to analyze chemotactic responses of indigenous microbial species in a non-invasive and label-free manner, non-labelled *Comamonas testosteroni* CNB-1 was selected and tested with 4-hydroxybenzoic acid (*p*-HBA) as its chemo-attractant. CNB-1 was isolated from sewage sludge of a wastewater-treatment plant from a chemical factory producing chloronitrobenzene^36^. Previous studies using swarm plate assays showed that CNB-1 exhibited strong chemotactic responses toward various aromatic compounds, including *p*-HBA^37^. Using the same setting as described above, droplets with an average volume of ~5.5 nL were generated after mixing with MSB media at the T-junction, and incubated at 30 °C for 24 h. The growth of non-labelled CNB-1 in droplets (Fig. 5C) was easily distinguished from those empty droplets (Fig. 5B) using bright-field microscopy. The percentage of droplets with non-labelled CNB-1 increased from 1.5% to 90.0% after exposure to *p*-HBA (Fig. 5D). This result represents increasing of λ value from 0.015 to 2.30 (about 150 times), indicating that CNB-1 was strongly attracted by *p*-HBA.

### Separate non-labelled chemotactic cells from a mixture

To demonstrate that this device can be utilized in enrichment of non-labelled species from mixtures based on chemotaxis, we mixed non-labeled CNB-1 and RFP-tagged *E. coli* RP437 at OD ratio of 1:10 (OD_CNB−1_ = 1.0, 2000 × dilution; OD_RP437_ = 1.0, 200 × dilution), and used *p*-HBA as the chemoeffector. We used the MSB medium for droplet cultivation with *p*-HBA as the sole carbon source. As both species have high motility, we tuned the flow rate at the waste outlet from 0.25 up to 0.30 μL/min, to reduce the ratio of RP437 in the enrichment. Other flow rates were the same as described above. After exposure to *p*-HBA, the ratio of droplets with CNB-1 increased dramatically from 3.6% to 27.1%. Whereas that of droplets with RP437 decreased from 8.0% to 1.7% (Fig. 5D). To exclude the coexistence of CNB-1 and RFP-tagged RP437 in droplets, we first determined λ values for the total population by bright-field enumeration, and then use the G-2 A filter to discriminate droplets containing RFP-tagged RP437 according to its fluorescence (Fig. 5A). The λ value of CNB-1 (λ_CNB−1_) was calculated by subtracting the average cell number per drop of RP437 (λ_Ref_) from the total (λ_total_). In the control experiments without *p*-HBA gradient, λ_CNB−1_: λ_Ref_ was 0.040: 0.084 = 0.48. This ratio increased to 0.298 : 0.017 = 17.76 when the sample was exposed to *p*-HBA (Fig. 5E). Therefore, we estimated that the chemotaxis toward *p*-HBA yielded a high enrichment factor of 36.7 times for CNB-1 against RP437 on this device.

## Conclusion

In summary, we have successfully developed a versatile microfluidic system for continuous sorting of chemotactic microbial cells, followed by high throughput single cell cultivation and enumeration in an array of nanoliter droplets. Single-cell cultivation in droplets allows facile evaluation of chemotactic responses of a microbial species toward different chemoeffectors, without relying on fluorescent labeling and real-time microscopic imaging. On the other hand, standard fluorescent-labelled strains can be used in our experiment, serving as reference for quality control, or as internal standards for non-labelled strains. Using this device, we separated chemotactic *E. coli* RP437 from its non-chemotactic mutant RP1616. Furthermore, non-labelled CNB-1 strain was successfully enriched from a mixture based on its distinctive chemotaxis toward aromatic compounds.

Similar to other parallel-flow chemotaxis assays, the current system cannot totally avoid interfering effects such as shear stress, rheotaxis^38^, and contamination of highly motile but non-chemotactic cells. To exclude these factors, it is important to have negative controls for all chemotaxis assays. It also should be noted that, the average number of cells per droplet (λ) can be more than 1 for reliable estimation of chemotactic response based on Poisson distribution^39^. Increasing the amount of droplets helps in improving the precision of the chemotaxis assays. However, to achieve a higher probability of single-cell cultivation, λ should be strictly controlled at a low level. For example, we have to decrease λ to 0.1 to obtain single-cell cultivation in 95% of growth-positive droplets, which means that more than 90% of droplets have to be empty.

Except providing quantitative chemotaxis analysis, this system may also be applied in assessing microbial viability and motility under physicochemical stresses^40^,^41^. Moreover, multiple species can be separated as single cells in droplets and directly recovered as isolates after incubation. Selective culture media can be used to enhance the growth of target species in droplets, as well as suppress the growth of contaminating species. As samples can be continuously processed and cultivated at the single cell level using this device, selective enrichment of rare chemotactic species from the environmental samples might be achieved. Directed selection of highly chemotactic mutant from a mixture can be realized by performing multiple cycles of chemotactic sorting and cultivation. The gas-permeable Teflon tubing is promising for isolation and cultivation of both anaerobic and aerobic species. We believe that with further integration and development, this system can be widely used in studying chemotaxis of microbial cells, and it can also be applied for discovery of functional microbial species from various environments.

## Materials and Methods

### Device fabrication

The polydimethylsiloxane (PDMS) microfluidic device was fabricated using soft lithography technique as described^42^. The top layer contains microchannel structures. Teflon tubing was cut at an angle and inserted from the outlet of the PDMS device up to the T-junction and sealed in place with capillary wax (Hampton Research, Aliso Viejo, CA).

### Device operation

All tubing, devices, and syringes used are sterilized by 70% (v/v) ethanol, and all solutions and media used are either autoclaved or filtered through a PES or PTFE filter with 0.22 μm pore size. Prior to experiment, the main channel for chemotaxis is selectively blocked with bovine serum albumin (10 mg/mL) to avoid the nonspecific adsorption of cells. Solutions are loaded into gastight syringes (Agilent, Reno, NV) with fixed 27 gauge needles and 30 gauge Teflon tubing (Zeus, Branchburg, NJ), and infused into the device by syringe pumps (Harvard Apparatus, Holliston, MA). All pumps except the one connected to the waste outlet infuse solutions into the device (Fig. 1B). The fluorinated oil FC-40 (3 M, St. Paul, MN, USA) was used as the carrier phase for droplet generation. Once the Teflon tubing is filled with droplets, it is detached from the device, sealed at both ends with wax, and the pumps are stopped. A new Teflon tubing can be attached to the device for another cycle of droplet collection. The Teflon tubing with the droplet array is placed in a Petri dish pre-filled with water, and incubated at 30 °C or 37 °C for cell growth.

### Preparation of microbial cells

*Escherichia coli* (*E. coli*) strains RP437 and RP1616 were kindly provided by Professor J. S. Parkinson from the University of Utah. *E. coli* RP437 was transformed with DsRedT.4 plasmid, while *E. coli* RP1616 was transformed with pACGFP1 plasmid (Clontech Laboratories, Mountain View, CA). *E. coli* strains were cultivated at 37 °C in Luria Bertani (LB) medium with 100 μg/mL ampicillin. CNB-1 was cultivated at 30 °C in mineral salt broth (MSB) as previously described^36^. All the cells in the seed culture were harvested at the exponential phase, centrifuged and suspended in 1 × PBS containing 0.1 mmol/L EDTA as the pre-sample. The numbers of live cells for each species were estimated by measuring optical density through a UV/Vis spectrophotometer (Metash, Shanghai, China). We used 10 mmol/L Asp in 1 × PBS (pH = 7.0) as the chemoeffector for chemotaxis of *E. coli* RP437, and 5 mmol/L *p*-HBA in 1 × PBS (pH = 7.0) for CNB-1.

### Imaging and data processing

Chemotactic migration of *E. coli* RP437 in the main channel was imaged with G-2 A filter cube (excitation: 535/50 nm; emission: 590 nm long-pass) on an inverted fluorescence microscope (Eclipse Ti, Nikon, Tokyo, Japan), equipped with a CoolSNAP HQ^2^ camera (Photometrics, Tucson, AZ). Fluorescein gradients were visualized using a FITC filter cube (excitation: 480/40 nm; emission: 527/30 nm). After incubation, droplets were imaged in G-2 A, FITC, bright-field or phase contrast mode. All images were processed with ImageJ (NIH, Bethesda, MD).

### Cell counting based on Poisson distribution

In *Part II*, chemotactic cells were mixed with the culture medium and segmented into nanoliter droplets, with microbial cells randomly encapsulated in droplets according to Poisson distribution (Fig. 3A)^30^,^43^. Some contain no cell, some contain one cell, and others contain more than one. The distribution of cells in droplets is given by


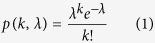


where *p* (*k*, *λ*) is the probability of *k* cell(s) per drop, and *λ* is the average number of cells per droplet. To quantify chemotactic responses of the bacteria, droplets were incubated to allow cell growth, and enumerated to determine the average number of cells per drop.

### Measure cell density with Poisson distribution

We prepared a sample of RFP-tagged RP437 with calibrated cell density of 1.3 × 10^7^ CFU/mL in 1 × PBS, and diluted it 10, 100, and 1000 times using 1 × PBS (pH = 7.0). To make the experiment simpler, we blocked all access holes on device in *Part I* (Fig. 1) except the inlet for loading cell suspension. The cell suspension was injected at a flow rate of 0.25 μL/min, and mixed with culture media, which flow rate was 0.3 μL/min. The flow rate for carrier oil FC-40 is 3.0 μL/min for generating droplets. Droplets were incubated to allow growth of cells. All droplets were imaged using the G-2 A filter on the inverted fluorescence microscope. Total number and number of droplets with cell growth were enumerated for performing the statistical analysis based on Poisson distribution.

## Additional Information

**How to cite this article**: Dong, L. *et al.* Automated Chemotactic Sorting and Single-cell Cultivation of Microbes using Droplet Microfluidics. *Sci. Rep.* **6**, 24192; doi: 10.1038/srep24192 (2016).

## Supplementary Material

Supplementary Information
