# Supplementary material for: Automated Chemotactic Sorting and Single-cell Cultivation of Microbes using Droplet Microfluidics
Source: Sci Rep. 2016 Apr 14;6:24192. doi: 10.1038/srep24192 (PMC4831006; doi:10.1038/srep24192)
Supplement: Supplementary Information [file srep24192-s1.pdf]

# Automated Chemotactic Sorting and Single-cell Cultivation of Microbes using Droplet Microfluidics

Libing Dong[1,2], Dong-Wei Chen[2], Shuang-Jiang Liu[2], Wenbin Du*[2]

[1] Department of Chemistry, Renmin University of China, Beijing 100872, China.
[2] State Key Laboratory of Microbial Resources, Institute of Microbiology, Chinese Academy of Sciences, Beijing 100101, China.

Correspondence and requests for materials should be addressed to W.D. (email: wenbin@im.ac.cn)

**Supplementary Figures:**

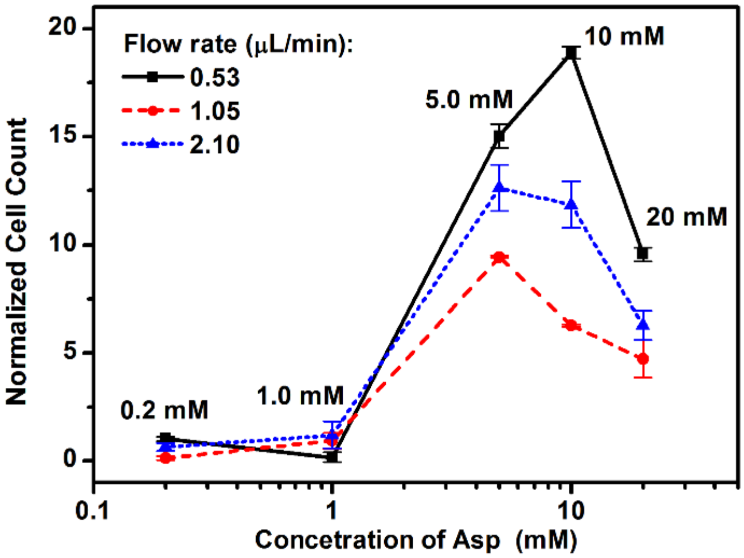


**Fig. S1** The effect of Asp concentrations on chemotaxis of RP437 in flow-based gradient evaluated based on real time imaging in the main channel.

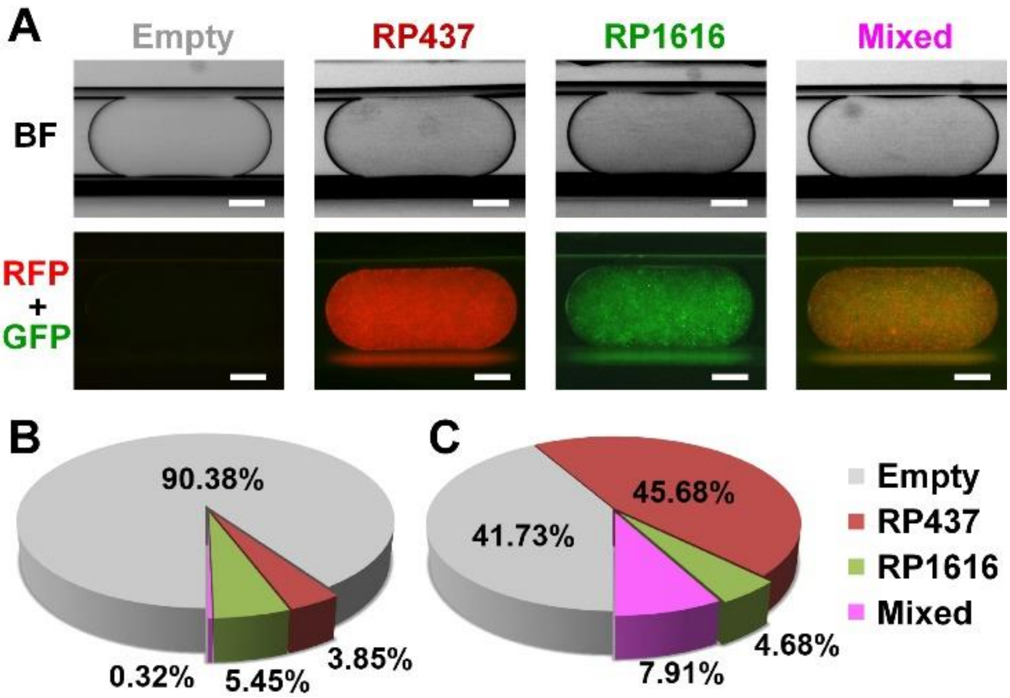


**Fig. S2** (A) Representative microscopic images for droplets after 24 h incubation. A mixture of *E. coli* RP437 and *E. coli* RP1616 were loaded on the device for chemotaxis with 10 mM aspartic acid (Asp) as the chemoeffector. Those chemotactic cells were collected and mixed with culture media for incubation. The first row is bright-field images; the second row is merged images of green and red fluorescence. Pie charts of the ratio of droplets classified into four types: only RP437, only RP1616, both, and empty before (B) and after (C) exposure to Asp. Scale bar = 200 µm.
